# An assessment of food supplementation to chronically sick patients receiving home based care in Bangwe, Malawi : a descriptive study

**DOI:** 10.1186/1475-2891-4-12

**Published:** 2005-03-21

**Authors:** Cameron Bowie, Linda Kalilani, Reg Marsh, Humphrey Misiri, Paul Cleary, Claire Bowie

**Affiliations:** 1 Department of Community Health, College of Medicine, University of Malawi, Blantyre, Malawi; 2 Johns Hopkins Research Project, College of Medicine, Blantyre, Malawi; 3 University of Auckland, New Zealand; 4 Department of Public Health, University of Manchester, UK; 5 Salvation Army Bangwe Project, Blantyre, Malawi

## Abstract

**Background:**

The effect of food supplementation provided by the World Food Programme to patients and their families enrolled in a predominantly HIV/AIDS home based care programme in Bangwe Malawi is assessed.

**Methods:**

The survival and nutritional status of patients and the nutritional status of their families recruited up to six months before a food supplementation programme started are compared to subsequent patients and their families over a further 12 months.

**Results:**

360 patients, of whom 199 died, were studied. Food supplementation did not improve survival but had an effect (not statistically significant) on nutritional status. Additional oil was given to some families; it may have improved survival but not nutritional status.

**Conclusion:**

Food supplementation to HIV/AIDS home based care patients and their families does not work well. This may be because the intervention is too late to affect the course of disease or insufficiently targeted perhaps due to problems of distribution in an urban setting. The World Food Programme's emphasis on supplementary feeding for these families needs to be reviewed.

## Introduction

The World Food Programme (WFP) has just launched a 328 million euro appeal to support 1.5 million people a month in five southern African countries including Malawi ravaged by the "triple threat" of food shortages, high HIV/AIDS rates and weakened capacity for governance [1]. A WFP document stresses the importance of food as "The First Line of Defence" in the fight against HIV/AIDS [2]. There are no reported trials evaluating the effect of food supplements on people in home based care programmes. However it seems plausible that food supplementation to such patients and their families might help prolong survival, help reduce wasting and alleviate symptoms [3]. Weight loss in such patients is usually due to a combination of nutrient malabsorption, changes in metabolism and reduction in food intake [4]. Such patients need more food not less but the latter situation becomes common when food security is a problem due to loss of income in urban areas and due to loss of manpower in rural areas [5]. Two small clinical trials have reported micronutrients reduce progression of AIDS in the early stages of disease [6,7].

The Bangwe project is a joint home based care (HBC) project run by the Salvation Army and the Department of Community Health, College of Medicine, University of Malawi. While providing a standard HBC service to the people of Bangwe, a township of 40,000 people adjacent to the city of Blantyre, the opportunity is taken to collect data on the health problems of patients, their response to treatment and their nutritional status. Antiretroviral drugs were not being used by any patient during the study period, which was a time before antiretroviral therapy became available free of charge in Malawi. The project is part funded by the National AIDS Commission (NAC) and partly by voluntary contributions and external donor NGOs to the Salvation Army.

The project has been collecting patient data since January 2003. The NAC suggested that the project could assess the use of supplementary feeding to HBC patients. WFP agreed to supply the food. Supplementary feeding started in July 2003. It was not known if oil should be included in the basic food package. Some oil was made available and the opportunity was taken to randomly allocate oil to half the patients. Nutritional assessments were carried out at the time of initial assessment of the patient and in June 2003, November 2003 and July 2004 on the members of the household of each patient.

## Methods

This is an observational "before and after" study using routinely collected data. It is not clinical research as such but is a description of outcomes following the introduction of food and, to some, oil supplementation.

The study area is four villages in Bangwe with an estimated population of 23,044 at the last census taken in 1998, and now thought to have grown to 26,500 based on a census carried out in part of the area in 2003 which found a population growth of 15% in the five years since the national census. Inclusion criteria are that patients are adults (over 15 years of age) with chronic disease of more than a month, and in need of home based care. Patients receive an initial assessment including height and weight measurements if fit enough to stand. Clinical data are collected at each follow up visit concerning the progression of symptoms. Anthropometric measurements have been made on three occasions of each household member of those patients alive in June 2003, in November 2003 and in July 2004. The group of patients enrolled between January and June 2003 and their families did not receive food in the first period of their home based care. They and all subsequent patients received the basic food package from July 2003 to July 2004.

The effect of food on nutritional status will be affected by the duration of the food and the disease. This is measured by the difference in nutritional status of each patient found comparing the body mass index (BMI) at the latest survey with the original BMI using the rate of change in BMI per 100 days.

The WFP food supplementation programme was targeted at households taking care of orphans and those with someone requiring home based care. Monthly rations were distributed:-

• 50 kg of maize

• 5 kg of beans

• 7.5 kg of Likuni Phala (cereal-soya blend)

In addition half the households received 4 litres of oil. Food distribution began in July 2003 and finished in September 2004.

Half the patients were randomly allocated to receive the supplementary oil. This allocation was not strictly adhered to in 2004. The opportunity was taken to ask patients if they had received oil or not, and if so for how many months, as part of the July 2004 nutrition follow up survey.

The patient information was recorded by project staff and entered onto a database using epi info and excel. Patients were categorised by presenting symptoms into one of the four clinical stages as used in a recent WHO staging system [8]. The study was divided into three periods; the first period was from the start in January 2003 to July 2003 which was the time of the first nutritional survey and just before food distribution began; the second period was from August 2003 to November 2003 which was when a second nutritional survey was undertaken; the third period from mid November 2003 to July 2004 which was the time of the third nutrition survey. Results were analysed using SPSS.

## Results

360 patients were enrolled between January 2003 and July 2004 of which 59% were women. Most patients have HIV/AIDS and less than 5% have other chronic conditions such as paraplegia. Not all eligible patients living in the study area are identified by community volunteers and others do not wish to receive the home based care service. A census of part of the area carried out in 2003 found 7.8 per 1000 aged between 13–49 years were chronically sick (for more than a month). This equates to 205 chronically sick of this age group living in the study area. At the time of the census 142 patients had been enrolled into the HBC service of which 97 were still alive. It appears that about half the chronic sick in the study area were enrolled at the time.

**Figure 1:**
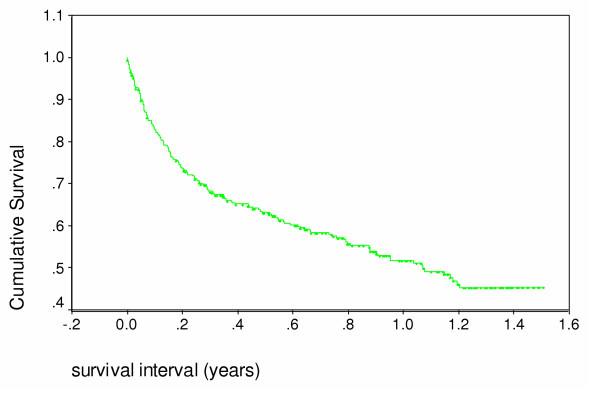
Survival of all home based care patients – Kaplan Meier analysis.

Of the 360 patients, one third died within 6 months (Figure 1). The median survival was 1.2 years up to July 2004. Since the start of the study 199 of the 360 patients have died (56%).

**Figure 2:**
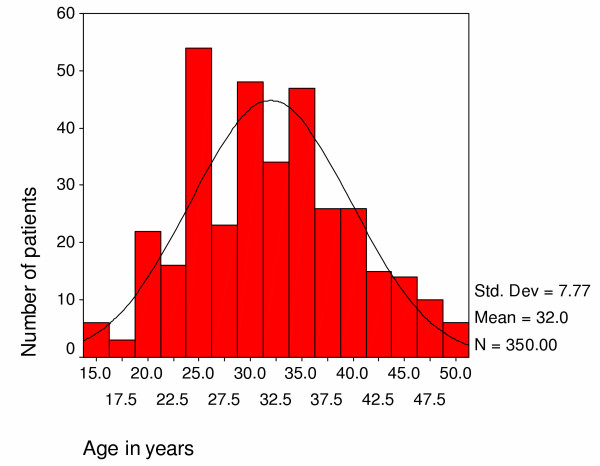
Age distribution of home based care patients.

**Figure 3:**
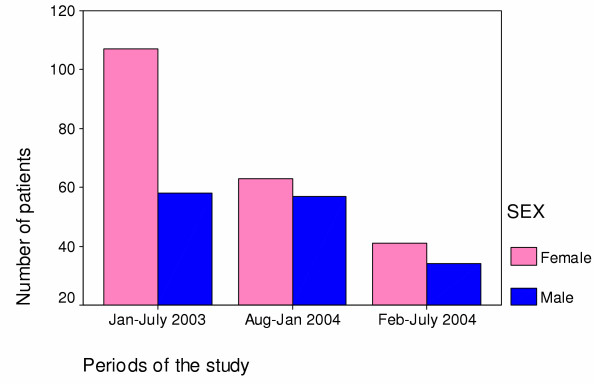
Sex distribution of patients enrolled in three periods of the Bangwe study.

The age distribution of patients follows one well recognised in AIDS patients with a mean age of 32 years (Figure 2). Females were younger (mean age of 30.9 years with standard deviation (SD) of 7.8) than males (mean age of 33.4 years with SD of 7.5). More females than males were enrolled in the first six months of the study (Figure 3). However there was no apparent difference in case severity of patients enrolled in the different periods of the study based on symptoms of fever, cough, lower limb pain and thrush using discriminant analysis (Wilks' Lambda Test = 0.98, p= 0.803), or body mass index (p = 0.63).

### Clinical stage at presentation

The majority of patients presented in an advanced stage of disease, with 70% in stage 4 (Table 1). None presented with stage 1 disease. There is no statistical difference in the stages of presentation in the three periods (Chi^2 ^= 7.2, df = 4, p = 0.13).

**Table 1:** Clinical staging by period of recruitment – Bangwe

	Clinical staging			Total
PERIOD	2	3	4	

1	14	40	111	165
2	3	30	87	120
3	1	17	46	64
Total	18	87	244	349
%	5%	25%	70%	100%

Chi sq = 7.2, df 4, p = 0.13

period 1 – patients recruited before food supplementation started

period 2 – patients recruited in the first six months after food supplementation started

period 3 – patients recruited in the second six months period after food supplementation started

### Nutritional status of patients

**Figure 4:**
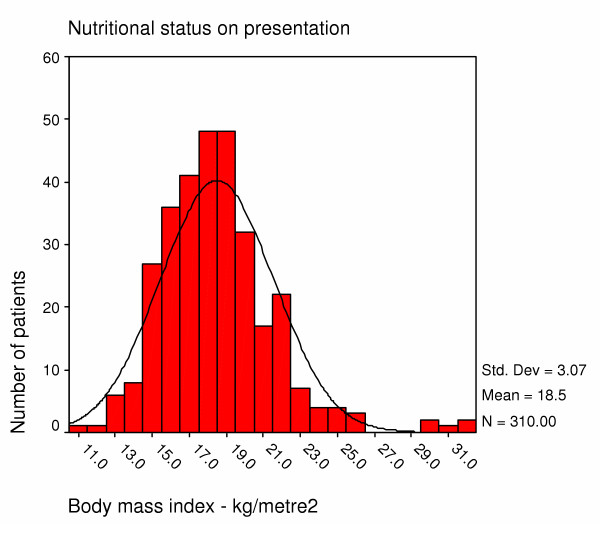
Nutritional status of home based care patients on first presentation.

The mean BMI at presentation was 18.5 kg/m^2 ^(SD 3.1). Half the patients were malnourished with a body mass index (BMI) of less than 18.5 kg/m^2 ^on enrolment. A quarter was severely malnourished below 16 kg/m^2 ^(Figure 4). The nutritional status of the group presenting before July 2003 is similar to that of the group presenting later (Table 2).

**Table 2:** Nutritional status of patients enrolled before and after start of food distribution

Period	Mean BMI (kg/m^2^)	Lower 95% CI	Upper 95% CI
Before July 2003	18.4	17.8	19.0
After June 2003	18.6	18.1	19.1

The three follow up nutrition surveys provide some evidence of the changing status of the patients who survived. No one received food before the first follow up survey in June 2003. During this time nutritional status remained constant (Table 3). In subsequent surveys the mean BMI of those still alive rises by 0.49 kg/m^2 ^per 100 days by the second survey and 0.46 kg/m^2 ^by the time of the third survey, not quite statistically significant (Anova – F = 2.58, df = 2, p = 0.08).

**Table 3:** Mean change in BMI per 100 days for patients who survived from initial assessment to one of three surveys in Bangwe

		Number	Mean	Std. Deviation	Std. Error	95% Confidence Interval for Mean	
						Lower Bound	Upper Bound

Change in BMI to June 2003		61	.07	1.64	.21	-.35	.49
Change in BMI to Nov 2003		70	.49	.82	.10	.29	.68
Change in BMI to July 2004		81	.46	.96	.11	.25	.67
Total		212	.36	1.17	.08	.20	.52
Model	Fixed Effects			1.16	.08	.20	.51
	Random Effects				.13	-.20	.91
ANOVA	Sum of Squares		df	Mean Square	F	Sig.	
Between Groups	6.95		2	3.48	2.58	.08	
Within Groups	281.96		209	1.35			
Total	288.91		211				

There is a small group of patients, 22 in number, who have survived through all three surveys (Table 4). For them there is an increase in the rate of change of BMI between the pre-food and the first post food period but not the second period (Wilks' Lambda = 0.84, p = 0.17, partial eta squared = 0.16).

**Table 4:** Rate of change of nutritional status of 22 patients who survived all three surveys

	Mean	Std. Deviation	N
change in BMI per 100 days from initial assessment to June 2003 survey	.30	1.80	22
change in BMI per 100 days from June to Nov 2003 surveys	.84	.92	22
change in BMI per 100 days from Nov 2003 to July 2004 surveys	.17	1.09	22

Wilks' Lambda = 0.84, p = 0.17, partial eta squared = 0.16

### Oil supplementation

The addition of oil to the food package has no effect on nutritional status as measured by a change in mean BMI per 100 days (Table 5).

**Table 5:** Comparison of change in nutritional status (change in BMI per 100 days) between those who did or did not receive oil as part of food supplementation

	oil actually received	Number	Mean	Std. Deviation	Std. Error Mean
rate of BMI change per 100 days from initial assessment to latest survey	OIL	50	.26	.67	.09
	NO OIL	14	.39	1.03	.27

Student t = -0.59, df = 62, p = 0.56

### Survival

**Figure 5:**
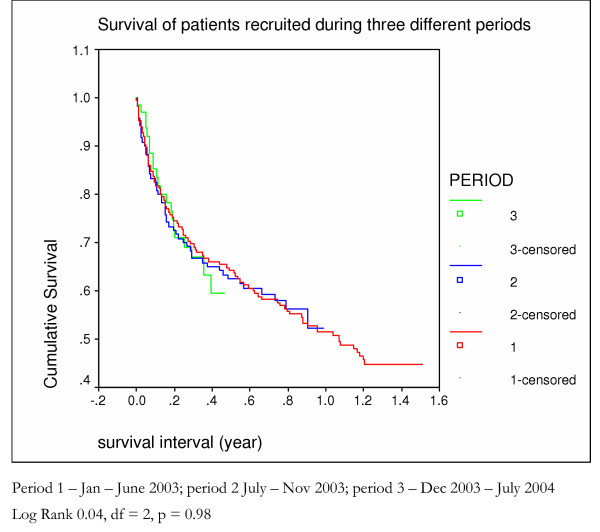
survival of patients enrolled in the home based care scheme during three different periods – Kaplan Meier analysis.

**Figure 6:**
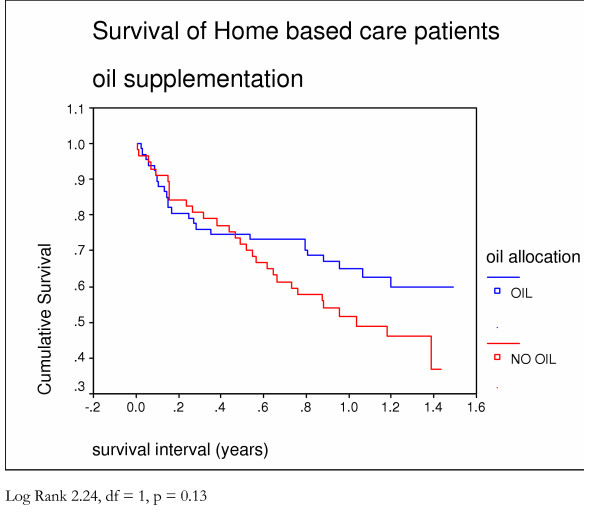
Survival of home based care patients who were or were not allocated oil supplementation – Kaplan Meier analysis.

**Figure 7:**
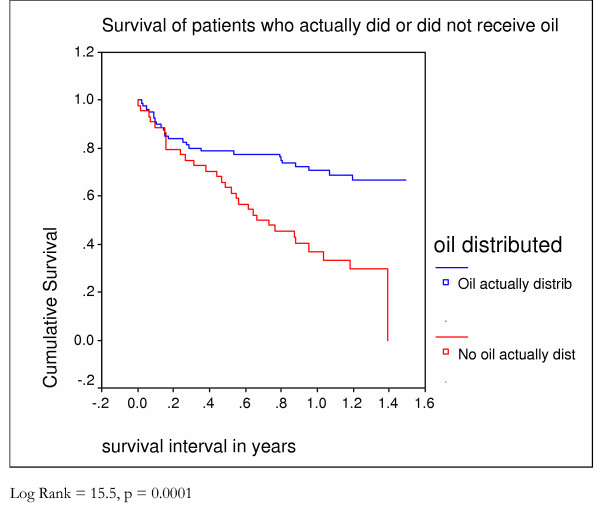
Survival of home based care patients who did or did not actually receive oil – Kaplan Meier analysis.

A third of patients died (112) within 4 months of being first seen. Half (180) survived fourteen months (Figure 1). There was no difference in the survival patterns of those who in the first months after presentation did not receive food compared to those who received food from the start (Figure 5). Survival was better in those **allocated **to receive oil (Figure 6) and those who **actually received **oil (Figure 7) compared to those who did not, although results are only statistically significant for those who actually received oil. Oil seems to have an effect but only for those who survive six months from time of initial assessment.

**Figure 8:**
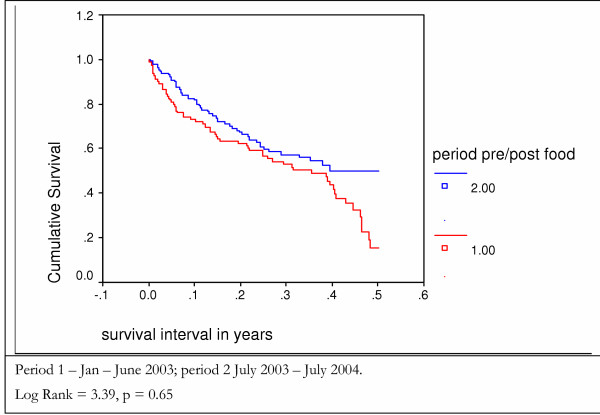
Survival of patients who presented in clinical stage 4 disease in the first sixth months after presentation – Kaplan Meier analysis.

**Figure 9:**
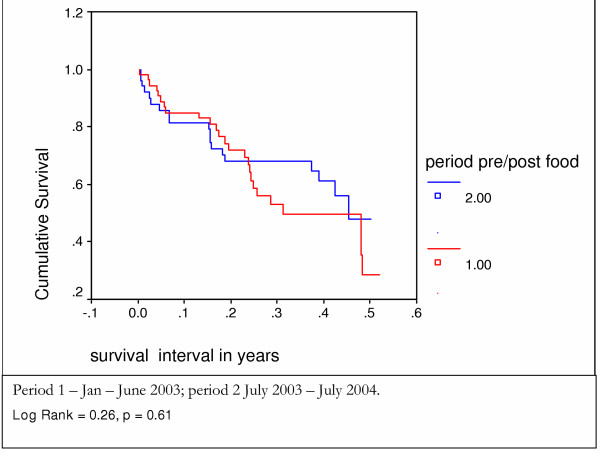
Survival of patients who presented in clinical stage 2 or 3 disease in the first six months after presentation – Kaplan Meier analysis.

The survival of Period 1 patients prior to receiving food can be compared to a group of patients in Period 2 over a similar 6 months period. The results show no statistically significant difference between the before and after food distribution groups, although there is a suggestion of improved survival in the after food group for clinical stage 4 patients (figures 8 and 9).

### Household nutrition

Households of patients enrolled between January and June who were measured in July 2003 were compared to households of patients measured in the July 2004 survey. Some of the families of patients who were enrolled before July 2003 and who survived a year were measured in 2004 and included in both groups. The mean BMI has fallen between the two measurement dates (Tables 6 and 7). This is despite food supplementation from July 2003. The pattern of a lower mean BMI of household members persists when those surveyed in 2003 are excluded from the "2004" group, and for different age groups.

**Table 6:** Mean Body Mass Index of households all ages comparing two groups, one surveyed (in June 2003) before and the other (in July 2004) after food distribution

	Group	N	Mean	Std. Deviation	Std. Error Mean
BMI	2003	506	21.86	6.54	.29
	2004	343	19.38	4.22	.23

Student t = 6.2, df = 847, p < 0.001

**Table 7:** Mean BMI of households of residents over 4 years of age (in June 2003) before and the other (in July 2004) after food

	Group	N	Mean	Std. Deviation	Std. Error Mean
BMI	2003	434	22.02	6.39	.31
	2004	290	19.65	4.32	.25

Student t = 5.5, df = 722, p < 0.0

## Discussion

An observational study of this sought provides clear outcome data based on the realities of the real world and not on the results of an artificially designed clinical trial. Food supplementation seems to have no effect on survival although it does on the nutritional status of those home based care patients who survive to one of the follow up weighing surveys. The result is not surprising considering the late stage in presentation of the disease in many patients. Another possible reason for the absence of effect on survival could that little food reaches the terminally ill patient due to problems of distribution in an urban area of Malawi to families who may have no one to carry food home from the distribution point and where many neighbours are hungry.

Oil, however, may have an effect in those patients who survive six months. This can be explained by some of the oil being eaten by the patient so providing a concentrated source of energy for those patients who are not terminally ill. An alternative suggestion is that oil is a saleable commodity and money so realised may be used to purchase essential commodities such as water and charcoal. This possible explanation fits with the result of oil having no demonstrable effect on nutritional status. There is a slight suggestion which is not statistically significant, as seen in the survival curves for clinical stage 4 patients that food may prolong survival after the first three months. No such difference is found in stage 3 patients.

Food supplementation has not helped to maintain body mass in household members of home based care patients. This apparently disappointing result needs interpretation. The reduction in mean BMI of household members may be attributable to the socio-financial catastrophe brought on by loss of income and increase in expenditure due to the chronic ill health of one or two of the adults in the family. The longer the adult remains alive and ill, the longer the loss of earnings, drain on resources and ensuing poverty. This may account for the reduction in BMI of PLWA households some of whose patients have survived for 12 months or more. Perhaps food supplementation has alleviated this tendency to malnutrition. It could have been worse without the food.

An observational study of this sort is difficult to interpret. Bias can confuse interpretation if the groups which are compared are not similar. The severity of case mix has been compared using discriminant analysis of the presence and severity of presenting symptoms. The before and after food groups have similar case mix. Their BMIs are similar. The main difference is the preponderance of females in the before-food group. It may be that males tend not to seek home based care until it is known that food is available. However, the severity of disease of these patients does not seem to be different from the severity of disease of those presenting before the food handouts started. It appears that the two groups at first presentation are comparable.

Disease progression without antiretroviral drugs is usually inevitable and insidious. Some patients who present with terminal illnesses require palliative care such as opiates and soon die. Others do stabilise with the majority needing continuous or intermittent treatment to provide palliation of symptoms. But should food supplementation be considered one such intervention?

The benefits of food, if they exist, may be outweighed by the costs, not just to donor organisations, but to the patients and their families. Food distribution in urban areas has problems and food may not get to the people intended. Indeed the WFP were hesitant about initiating the programme because of the problems likely to be encountered in Bangwe. The social disruption and animosities produced by the free but selective distribution of food in a community with slender food security may be substantial. The difficulty of families where the adults are unable to get out of the house to collect the food is real. Food is only one of the needs of the family. The catastrophe brought on by terminal illness in one or both caring adults is economic not famine. A more direct help would be the replacement of lost income. It is money in an urban area which is wanted, and not just food. Money is easier to distribute and replaces the actual loss experienced by such a family. The WFP has been reviewing the place of non-food responses to food insecurity and the value of targeting food insecure households [9]. Their review notes the lack of empirical experience of non-food aid interventions.

The possible role of oil in increasing survival requires further study. Not only does oil provide highly concentrated energy but it is also far easier to distribute to PLWA families. Oil is also an easily saleable commodity, with the income from such sales available to purchase other necessities. Two small trials in Bangkok and Tanzania suggest that vitamins may delay progression of AIDS. While a large scale trail is needed to confirm the potential of this, one possibility is to dispense ready to use food (RTUF) which has been found to be useful in the treatment of severely malnourished children in Malawi [10]. The locally produced high energy, high protein, vitamin fortified food which does not need cooking or keeping cool may be an ideal preparation to dispense with other palliative care drugs.

## Conclusion

Food supplementation to home based care patients does not seem to be effective despite the fact that many of the patients are severely malnourished. Those who present reasonably early in the disease and survive at least six months seem to benefit from the oil. The distribution of food supplementation to PLWAs in the late stages of disease may be ineffective. Household members of home based care patients do not seem to benefit from the food handouts. A possible explanation is that the food did not reach the mouths of the patients or their families due to problems of distribution in an urban area. The WFP emphasis on supplementary feeding for these families needs to be reviewed.

## Competing interests

The author(s) declare that they have no competing interests.

## Authors' contributions

CB conceived the survey and analysed and wrote the first draft. PC designed the survey and undertook preliminary analysis. CTB undertook and supervised the data collection. LA supervised data entry. RM and HM provided statistical support. All contributed to the final report.
